# Complete genome sequence of *Synechocystis* sp. LKSZ1 isolated from a pond in the Suzukakedai campus of Tokyo Institute of Technology

**DOI:** 10.1128/mra.00549-24

**Published:** 2024-09-09

**Authors:** Leonardo Ken Okumura, Shinji Masuda

**Affiliations:** 1 Department of Life Science and Technology, Tokyo Institute of Technology, Yokohama, Japan; SUNY College of Environmental Science and Forestry, Syracuse, New York, USA

**Keywords:** cyanobacteria, genomes, *Synechocystis*, PacBio, B-assembler, complete genome sequence, *Synechocystis* sp. LKSZ1, DFAST

## Abstract

Cyanobacteria belonging to the *Synechocystis* genus have been isolated from diverse aquatic environments. This announcement reports the complete genome sequence of *Synechocystis* sp. LKSZ1 newly isolated from a pond in a university campus in Yokohama, Japan. The genome sequencing was performed using the PacBio Revio HiFi long-read technology.

## ANNOUNCEMENT

Cyanobacteria are oxygenic phototrophic bacteria that inhabit diverse aquatic environments and play important ecological roles as primary producers (1). We have attempted to isolate cyanophages and their hosts from a pond in the Suzukakedai campus of the Tokyo Institute of Technology in Japan. Here, we report the complete genome sequence of *Synechocystis* sp. LKSZ1 isolated in the campus as a candidate host of cyanophages.

*Synechocystis* sp. LKSZ1 was isolated from a water sample of a pond at coordinates 35°30′53.6″ N 139°29′5.7″ E in December 2022. The isolation was conducted by plating, followed by colony pick and re-streaking eight times using BG11 (2) agar plates at 34°C under continuous light exposure (15–20 µmol photons m^−2^ s^−1^).

Genomic DNA isolation of the bacterium grown in a BG11 liquid medium was performed using a modified phenol–chloroform protocol as described on protocol.io (https://dx.doi.org/10.17504/protocols.io.rm7vzjyn8lx1/v1). Further purification of the genomic DNA was conducted using the NucleoSpin Microbial DNA Kit (Takara Bio Inc., Shiga, Japan; Takara Code: U0235A). The purified genomic DNA was used for whole genome sequencing, which was commissioned by Bioengineering Lab. Co., Ltd. (Kanagawa, Japan). The sequencing library preparation, including DNA shearing and size selecting, was conducted with the PacBio protocol (PN 101-987-800). Sequencing analysis was conducted with the Revio Polymerase Kit (Pacific Biosciences of California, Inc., Menlo Park, CA, USA) and PacBio Revio (Pacific Biosciences of California, Inc., Menlo Park, CA, USA). Data analysis was performed using SMRT Link (version 13.0.0.207600) to remove overhang adapter sequences from the reads, producing subreads that were aligned to generate consensus sequences (CCS). CCS reads with an average quality value below 20 were discarded, and the remaining reads were classified as HiFi reads after removing ultra-low PCR adapters using Lima software (version 2.7.1). The aforementioned process yielded 137,227 HiFi reads with an average length of 9,086 bp. Of these, 95.8% achieved a quality score of at least 99.90%. These reads were then used for further analysis. The raw reads were assembled into a predicted genome size of 3.4 Mbp using B-assembler, a tool for the circular bacterial genome assembly (3), resulting in a single circular genome. The assembled genome was assessed using BUSCO v5.7.0 (4) with the -m genome --auto-lineage-prok modes, utilizing the cyanobacteria_odb10 data sets and achieving 99.1% complete BUSCOs. Genome annotation was performed using DFAST v. 1.6.0 (5, 6). Genome statistics are summarized in Table 1 (Note: The GC percent is reported as 50.5% in Accession: ASM4043631v1 because NCBI rounds the numbers to 0.5% or the whole number). Default parameters were used for all software unless specified otherwise.

**TABLE 1:** The genome statistics for the cyanobacterium *Synechocystis* sp. LKSZ1

Total sequence length (bp)	3,671,396
Genome coverage	304 ×
GC content (%)	50.7
Number of CDSs	3,285
Average protein length	331.7
Coding ratio (%)	89.0
Number of rRNAs	4
Number of tRNAs	44
Number of CRISPRs	2

The 16S rRNA sequence from the genome (Accession: LC832711) was analyzed using BLAST+ 2.15.0 (7), revealing a 99.31% identity with *Synechocystis* sp. CCALA 700 (Accession: GQ375043.1). Additionally, the average nucleotide identity (ANI) was assessed with pyani v0.2.7 (8), comparing the assembled genome of LKSZ1 (Accession: ASM4043631v1) with that of PCC7339 (Accession: ASM1992409v1), resulting in an ANI of 73%. Moreover, GTDB-Tk v2.4.0 (9) analysis classified the genus as *Synechocystis*. These results confirm that the isolated bacterium belongs to *Synechocystis* sp., and we designated the strain as LKSZ1.

## Data Availability

The genome sequence of *Synechocystis* sp. LKSZ1 has been deposited in DDBJ/ENA/GenBank under the accession number AP031572. The associated BioProject has been assigned the accession number PRJDB18039. The raw sequencing data are publicly accessible via the DDBJ Sequence Read Archive under run accession number DRR551727. The accession number of the 16S rRNA sequence of the strain is LC832711. The assembled genome of *Synechocystis* sp. LKSZ1 (Accession: ASM4043631v1) was used for GTDB-Tk analysis and pyani analysis. The assembled genome of *Synechocystis* sp. PCC 7339 (Accession: ASM1992409v1) was also used for the latter analysis.
